# Magnetic Fe_3_O_4_@graphene oxide improves the therapeutic effects of embryonic stem cells on acute liver damage

**DOI:** 10.1111/cpr.13126

**Published:** 2021-09-27

**Authors:** Tahereh Foroutan, Mohammad Zaman Kassaee, Mahdi Salari, Fatemeh Ahmady, Fatemeh Molavi, Fariborz Moayer

**Affiliations:** ^1^ Department of Animal Biology Faculty of Biological Sciences Kharazmi University Tehran Iran; ^2^ Department of Organic Chemistry Tarbiat Modares University Tehran Iran; ^3^ Department of Environmental Health Engineering School of Public Health Hamadan University of Medical Sciences Hamadan Iran; ^4^ Faculty of Veterinary Medicine Islamic Azad University Karaj Iran

**Keywords:** acute liver failure, angiogenesis, conditioned medium, magnetic graphene oxide, stem cell therapy

## Abstract

**Objective:**

Acute liver failure is usually associated with inflammation and oxidation of hepatocytes and has high mortality and resource costs. Mesenchymal stem cell (MSCs) has occasionally been reported to have no beneficial effect due to poor transplantation and the survival of implanted cells. Recent studies showed that embryonic stem cell (ESC)‐derived MSCs are an alternative for regenerative medicine. On the other hand, graphene‐based nanostructures have proven useful in biomedicine. In this study, we investigated whether magnetic graphene oxide (MGO) improved the effects of ESC‐MSC conditioned medium (CM) on protecting hepatocytes and stimulating the regeneration of damaged liver cells.

**Materials and methods:**

To provide a rat model of acute liver failure, male rats were injected intraperitoneally with carbon tetrachloride (CCl_4_). The rats were randomly divided into six groups, namely control, sham, CCl_4_, ESC‐MSC‐CM, MGO and ESC‐MSC‐CM + MGO. In the experimental groups, the rats received, depending on the group, 2 ml/kg body weight CCl_4_ and either ESC‐MSC‐CM with 5 × 10^6^ MSCs or 300 μg/kg body weight MGO or both. Symptoms of acute liver failure appeared 4 days after the injection. All groups were compared and analysed both histologically and biochemically 4 days after the injection. Finally, the results of ESC‐MSC‐CM and MSC‐CM were compared.

**Results:**

The results indicated that the use of MGO enhanced the effect of ESC‐MSC‐CM on reducing necrosis, inflammation, aspartate transaminase, alanine aminotransferase and alkaline phosphatase in the CCl_4_‐induced liver failure of the rat model. Also, the expression of vascular endothelial growth factor and matrix metalloproteinase‐9 (MMP‐9) was significantly upregulated after treatment with MGO. Also, the results showed that the ESC‐MSC‐CM has more efficient effective compared to MSC‐CM.

**Conclusion:**

Magnetic graphene oxide improved the hepatoprotective effects of ESC‐MSC‐CM on acute liver damage, probably by suppressing necrosis, apoptosis and inflammation of hepatocytes.

## Graphical abstract

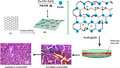

## 1 INTRODUCTION

Acute liver failure (ALF) is a life‐threatening clinical syndrome characterized by rapid hepatocellular necrosis due to hepatotoxicity, viral infection and immune‐mediated attacks.^1^, ^2^ It has high mortality and resource costs.^3^ Liver transplantation has some limitations such as the lack of donors, costs and immunosuppressive complications.^4^ Mesenchymal stem cells (MSCs) have also been used to treat ALF. They are easy to obtain and do not pose an ethical problem.^5^ MSCs have functions such as secretion of growth factors, angiogenesis and immunosuppression, as well as properties such as anti‐inflammatory and anti‐apoptosis effects. They can also prevent hepatocyte cell death and stimulate the regeneration of liver cells by paracrine mechanism or direct differentiation.^2^, ^6^

In view of the ALF treatment with stem cells, the available data are somewhat contradictory. Although MSC injection has some benefits,^7^, ^8^, ^9^, ^10^, ^11^ it has occasionally been reported to have no beneficial effect due to poor transplantation and the survival of implanted cells.^12^, ^13^, ^14^ In addition, the failure of MSCs to adhere to the target tissue leads to apoptosis of MSCs.^14^ However, limitations to harvesting MSCs from adult tissue include the invasive procedures in most cases, availability of suitable donors, limited number of cells obtained during the harvesting process, and restricted *in vitro* expansion capacity.^15^ Recently, it has been shown that MSCs could be derived from ESC, with similar phenotypic characteristics, along with favourable immunomodulatory and antiinflammatory properties that make them attractive candidates for regenerative medicine^15^, ^16^ and they have been used to treat various animal models disease such as experimental autoimmune encephalitis.^17^, ^18^, ^19^ On the other hand, administration of MSC‐conditioned medium (ESC‐MSC‐CM) has been shown to improve tissue injury.^20^, ^21^

The use of modern biotechnologies such as stem cells, laser and nanomaterial has attracted a great deal of attention in medical applications such as regenerative medicine.^22^, ^23^, ^24^, ^25^, ^26^, ^27^, ^28^, ^29^, ^30^ Graphene and its derivatives have been shown to improve the proliferation and differentiation of stem cells.^12^, ^13^ Due to their physicochemical properties and biocompatibility, sometimes they act as natural extracellular matrices (ECM) and are able to regulate the differentiation of stem cells.^31^

It has already been shown that the degree of cell differentiation and proliferation under different graphene oxide derivatives are different. For example, although in the absence of any osteogenic inducers the graphene nanogrids showed slight patterned osteogenic differentiations, the graphene sheets could not present any differentiation. On the other hands, the highly improvement of differentiation on the reduced graphene oxide nanoribbon grid was assigned to both its excellent effects on adsorption of the chemical osteogenic inducers and physical properties such as stresses oxidative.^32^, ^33^

Graphene oxide (GO) as a new class of carbon‐based nanomaterials is a derivative of graphene with a two‐dimensional honeycomb structure. The main difference between graphene and GO is the controllable hydrophilic nature of GO, which makes it well‐dispersible in water.^34^ Because of its small size, ease of use and large specific surface area, ^35^GO has been recommended for biomedical applications such as biosensors,^36^, ^37^, ^38^drug/gene delivery,^39^, ^40^, ^41^, ^42^and antibacterial effects.^43^, ^44^, ^45^ In addition, GO can absorb surface factors including proteins and small molecules, which are the essential components for the differentiation of MSCs.^46^, ^47^ However, it is difficult to separate GO from the solution by conventional centrifugation and filtration methods as a result of its hydrophilicity, high dispersibility and small size.^34^

Fe_3_O_4_ superparamagnetic nanoparticles are used in magnetically assisted drug delivery.^48^ They can easily be separated by a magnetic field.^49^, ^50^, ^51^, ^52^, ^53^ Nanocomposites based on magnetic graphene oxide (MGO) possess unique properties; they have high specific surface area, surface‐active sites, excellent magnetic characteristics, high chemical stability, an adjustable size and shape, and can be simply functionalized or modified.^34^The biocompatibility and anticancer effects of Fe_3_O_4_ in combination with graphene oxide have already been shown.^54^

In this study, we investigated whether the synthesized MGO increases the protective effects of ESC‐MSC‐CM in the treatment of liver damage in animal models. We studied the possible *in vivo* hepatoprotective effects of MGO mixed with ESC‐MSC‐CM on the rat model of acute liver failure induced by carbon tetrachloride (CCl_4_). CCl_4_ is a well‐known hepatotoxin and is often used to induce acute liver failure.^21^ The ability of ESC‐MSC‐CM mixed with MGO in the treatment of acute liver failure in rats was analysed by evaluating the serum level of enzymes and histopathological parameters.

## 2 MATERIALS AND METHODS

In this study, the GO and magnetic GO were produced according to Hummers et al. and Kassaee et al.^55^, ^56^ The GO prepared from graphite and the Fe_3_O_4_@GO composites were obtained by co‐precipitation of FeCl_3_·6H_2_O and FeCl_2_·4 H_2_O, in the presence of graphene oxide. The temperature was raised to 85°C, and ammonia solution was added for increasing the pH to 10. A mixture of styrene and benzoyl peroxide was added under a stream of N_2_ in 10 min. The final mixture was homogenized by sonication at 0°C for another 30 min. Polymerization was carried out by increasing the temperature of the mixture in an oil bath to 80°C for 4 h under an inert nitrogen blanket.

### 2.1 Characterization

GO and MGO were characterized by X‐ray diffraction (XRD, Philips Xpert MPD, Co K irradiation, = 1.78897A), Raman spectroscopy, scanning electron microscope (SEM, Philips XL30 microscope with an accelerating voltage of 25 kV), vibrating sample magnetometer (VSM), energy‐dispersive X‐ray spectroscopy (EDX), dynamic light scattering (DLS), Z potential and transmission electron microscopy (TEM) (PHILIPS, EM208S, Netherlands, at 100 kV of acceleration voltage.

#### 2.1.1 Animals and experimental design

Syngeneic male Sprague‐Dawley rats (220–280 g, Royan Institute) were kept under standard conditions in a light environment with temperature and humidity control. This study was approved by the Animal Care and Use Committee of the School of Biological Sciences at Kharazmi University. The acute liver damage model was obtained using a single intraperitoneal injection of 2 ml/kg body weight of CCl_4_ dissolved in sterile olive oil (1:1). Human ESCs line RH6 obtained as gift maintained in an undifferentiated state on mitomycin C‐treated mouse embryonic fibroblasts. Cells were subcultured on Matrigel‐coated plates in ESC medium. The cells were passaged every 5 days. It was derived human ESC‐MSC lines from RH6 ESC line, according to Hwang et al. protocol.^57^ To produce MSC‐CM, passage‐3 MSC cells were grown to 80% confluence cultured in a Dulbecco's modified eagle's medium supplemented with bovine serum albumin. MSC‐CM was collected after 10 h.

The rats were divided into six groups, namely a control group receiving no treatment, a sham group injected with only olive oil, a group injected with CCl_4_, a group injected with CCl_4_ and 400 μl ESC‐MSC‐CM of 5 × 10^6^ cells, a group injected with CCl_4_ and 300 µg/kg body weight of MGO and a group injected with CCl_4_, ESC‐MSC‐CM and 300 µg/kg body weight of MGO. All injections were intraperitoneal. For the sake of brevity, we refer to these groups as control, sham, CCl_4_, ESC‐MSC‐CM, MGO and ESC‐MSC‐CM + MGO, respectively.

The number of rats in each group was *n* = 6. All animals received treatment on the first day. They were anaesthetized with ether 4 days after the intraperitoneal injection of CCl_4_, ESC‐MSC‐CM and 300 µg/kg body weight of MGO. Blood samples were collected from the heart for biochemical analysis, and the livers were then taken for histological and immunostaining examination.

#### 2.1.2 Quantification of serum biochemical

The blood samples were kept at room temperature for 1 h and then centrifuged at 1500 *g* for 12 min at 4°C. The serum was separated and kept at 20°C until analysis. Serum levels of alanine aminotransferase (ALT), aspartate aminotransferase (AST) and alkaline phosphatase (ALP) were measured using an automated analyser (Hitachi) and commercially available kits (Pars Azmun) according to the manufacturer's instructions.

#### 2.1.3 Liver histology and immunohistology

The liver tissues were stained with haematoxylin–eosin (H&E) and observed with light microscopy (Zeiss). Primary mouse antibodies anti‐MPO (1/100; Abcam), anti‐CD68 monoclonal antibody (1:200; Serotec), anti‐MMP‐9 and anti‐ vascular endothelial growth factor(VEGF) were used to target neutrophils, Kupffer cells and liver regeneration‐related proteins, respectively. Anti‐TNF‐α, anti‐Caspase‐3 and anti‐IL‐6 were also used to identify apoptosis and proinflammatory cytokines.

#### 2.1.4 Vascular endothelial growth factor, TNF‐α and IL‐6 enzyme‐linked immunosorbent assay

Serum levels of VEGF, TNF‐α and IL‐6 were measured by ELISA method. They were measured with ELISA Kits Rat VEGF (RRV00; R&D; Inc), Rat TNF‐α (Dy510; R&D; Inc) and Rat IL‐6 (R6000B; R7D; Inc).

#### 2.1.5 Statistical analysis

The data were analysed using one‐way analysis of variance for multiple comparisons. The significance level was set at *p* < 0.05 and *p* < 0.01. All data are expressed as means ± *SE*. Kolmogorov–Smirnov test was used to study normal distribution. Parametric continuous data with normal distribution between different groups were compared by one‐way analysis of variance (ANOVA) followed by post hoc, Tukey test.

## 3 RESULTS

### 3.1 Synthesis of MGO nanohybrid

**FIGURE 1:**
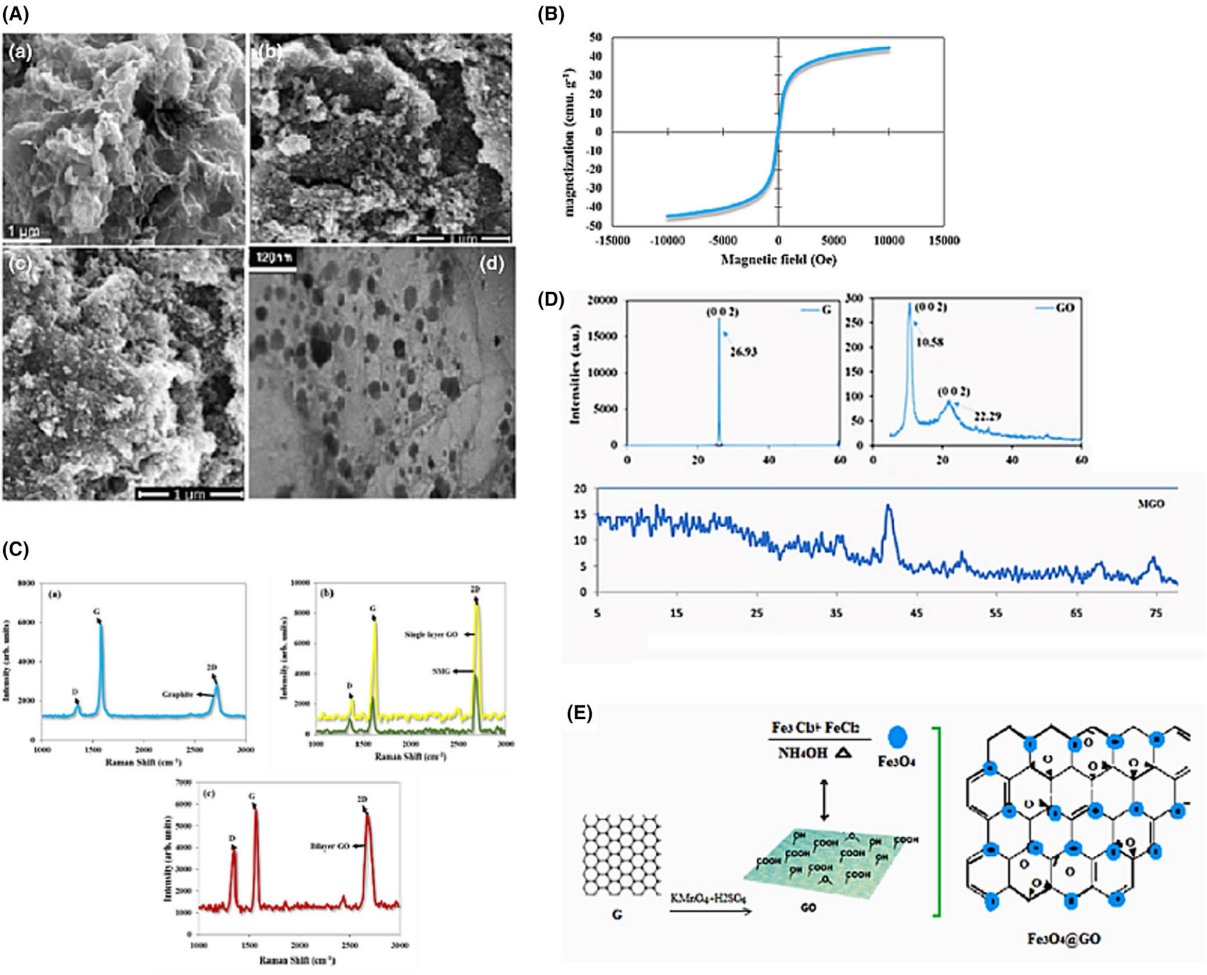
(A) SEM images of graphene oxide (GO) (a), magnetic GO (MGO) (c, d). TEM image of MGO (d). (B) and (C) are magnetic hysteresis loop of MGO and Raman spectra from graphite (a), single‐layer GO (b) and MGO (c), and bilayer regions of GO, respectively. XRD patterns of pristine graphite powder, GO and MGO (D). Schematic representations of the synthesis of graphite, GO and MGO (E)

Scanning electron microscopy (SEM) image of typical morphological features is depicted in Figure 1. As seen, the as‐prepared GO confirmed the synthesis of GO nanosheets with quite smooth surface, where the distinctive layered structure of the sample was evident. A sharp diffraction peak for the pristine graphite (2θ = 30.93°, index of 002, θ the Bragg angle, JCPDS No. 00‐012‐0212) is corresponding to a d‐spacing of 0.337 nm. The observation is in consistent with the previous study ^58^ The as‐prepared GO showed a characteristic peak at 2θ = 10.58° which resulted from the diffraction on its 002 layer together with a broad diffraction peak at 22.38°. Similar results have been reported in the previous study.^59^ The results for DLS and Zeta potential for pristine GO nanosheets and other nanoparticles analyses are given in Table 1. Figure 1f displays the magnetic hysteresis curve of MGO to characterize the magnetic properties of the composite. The result emphasizes a great paramagnetic behaviour of MGO, showing a saturation magnetization of 44.7 emu/g.^60^

**TABLE 1:** Results for DLS and zeta potential for pristine GO nanosheets and other nanoparticles

**Sample**	**Mean particle size (nm)**	**PDI**	**Zeta potential (mV)**
GO nanosheets	456.9	0.471	−51.8
MGO	584	0.214	3.9

Raman spectroscopy technique is applied to study structural characterization of carbon‐based material. The structural changes during the chemical reactions of carbon‐based material can be monitored by this technique. As seen in Figure 1g(a), Raman spectrum of graphite has obvious peaks displaying the D, G and 2D bands of graphite.^61^ Figure 1(b) shows the Raman spectra of single‐layer graphene oxide, displaying the G band at ∼1580 cm^−1^, the D band at ∼1350 cm^−1^ and the 2D band at ∼2679 cm^−1^. It is observed in Figure 1g(c) that in multi‐layer graphene sheets, the position of the G shifts into lower wavenumbers and 2D bands shifts into higher wavenumbers.^62^, ^63^ Figure 1g(b) also depicts the Raman spectrum of SMG composite. The position of bands shows no shift; however, the intensity of the bands has decreased. The similar result was found in the study conducted by Ning et al.^64^ The EDX analyses are given in Table 2.

**TABLE 2:** EDX results for pristine GO nanosheets and the other nanohybrids

**Sample**	**C (wt %)**	**O (wt %)**	**O/C**	**Fe**
GO nanosheets	54.43	45.12	0.79	−
MGO	23.00	35.83	1.59	48.17

### 3.2 MGO inhibits the release of liver enzymes and improves survival rate

**FIGURE 2:**
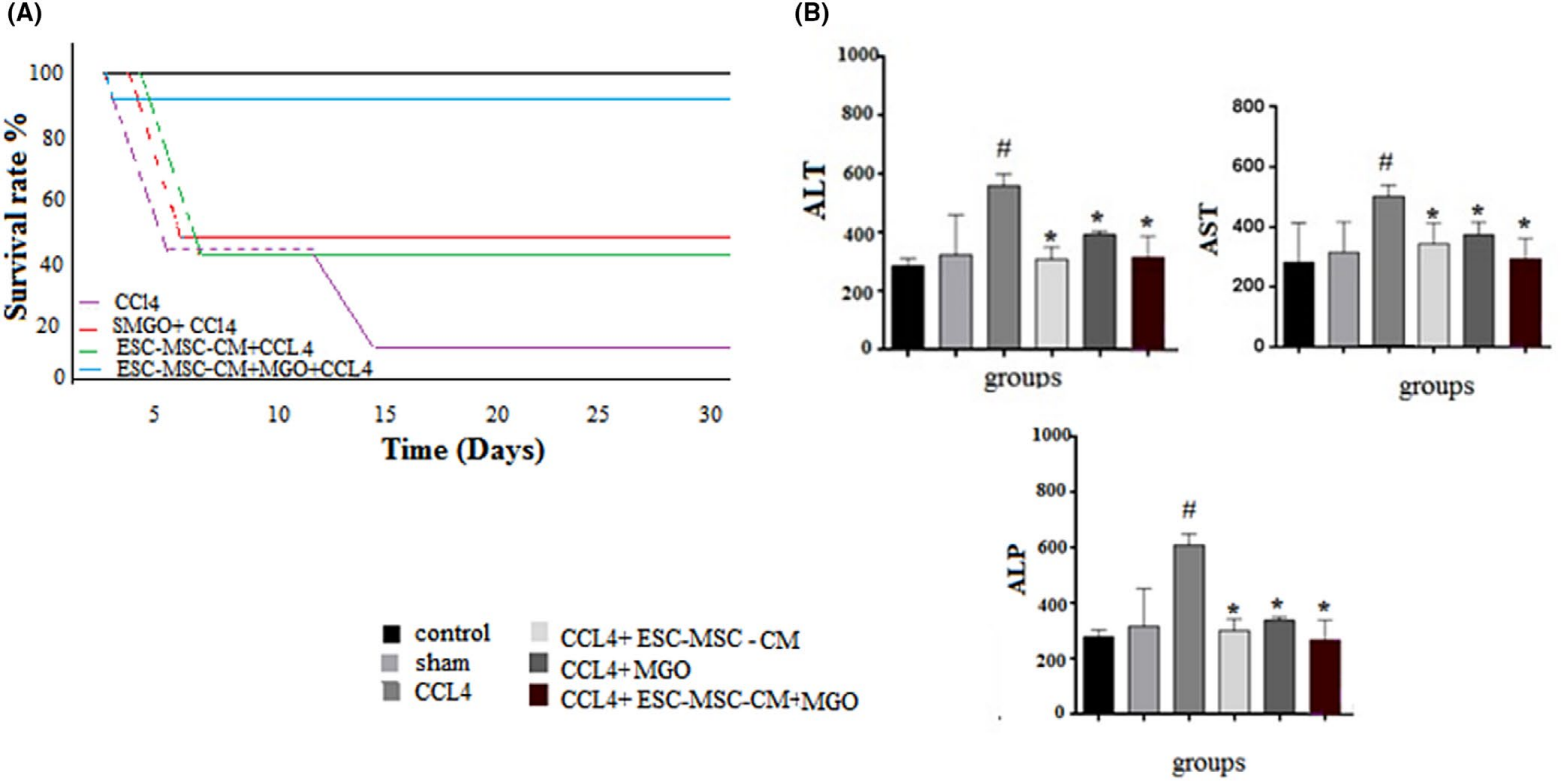
Treatment with MGO mixed with ESC‐MSC condition medium increased the survival rate (A), and decreased AST, ALT and ALP (B) serum levels of CCl_4_‐induced acute liver damage in rats collected 96 h after treatment (*n* = 5, *p < 0.05, ^#^ *p* < 0.01). Here, nano refers to MGO. Data are presented as mean ± SD. (*) and (^#^) indicate differences from the CCl4 and control groups, respectively, as determined by ANOVA with Tukey's HSD test. Abbreviations: ALT, alanine aminotransferase; AST, aspartate aminotransferase, ALP, alkaline phosphatase, CCl_4_, carbon tetrachloride; CM, conditioned medium; CCL4, carbon tetrachloride; MGO, magnetic graphene oxide

Our results indicated that treatment with MGO significantly improved the survival rate of CCl_4_‐induced liver failure in rats that received ESC‐MSC‐CM (Figure 2a). While only 20% of the rats survived for 35 days in the CCl_4_ group, 60% and 57% survived in the ESC‐MSC‐CM and MGO groups, respectively, and 100% survived during this period in the ESC‐MSC‐CM + MGO group. Liver damage was reduced in both ESC‐MSC‐CM and MSC‐CM + MGO groups compared to the CCl_4_ group 96 h after the injection. The recipients of ESC‐MSC‐CM + MGO developed liver dysfunction with significantly lower ALT, AST and ALP liver enzyme levels compared to the groups that received either ESC‐MSC‐CM or MGO (Figure 2b). Therefore, intraperitoneal injection of ESC‐MSC‐CM + MGO provided a significant survival benefit as it helped protect against liver damage and reduce mortality in recipient animals.

### 3.3 MGO injection improves histopathologic appearance and reduces neutrophil and Kupffer cell infiltration

**FIGURE 3:**
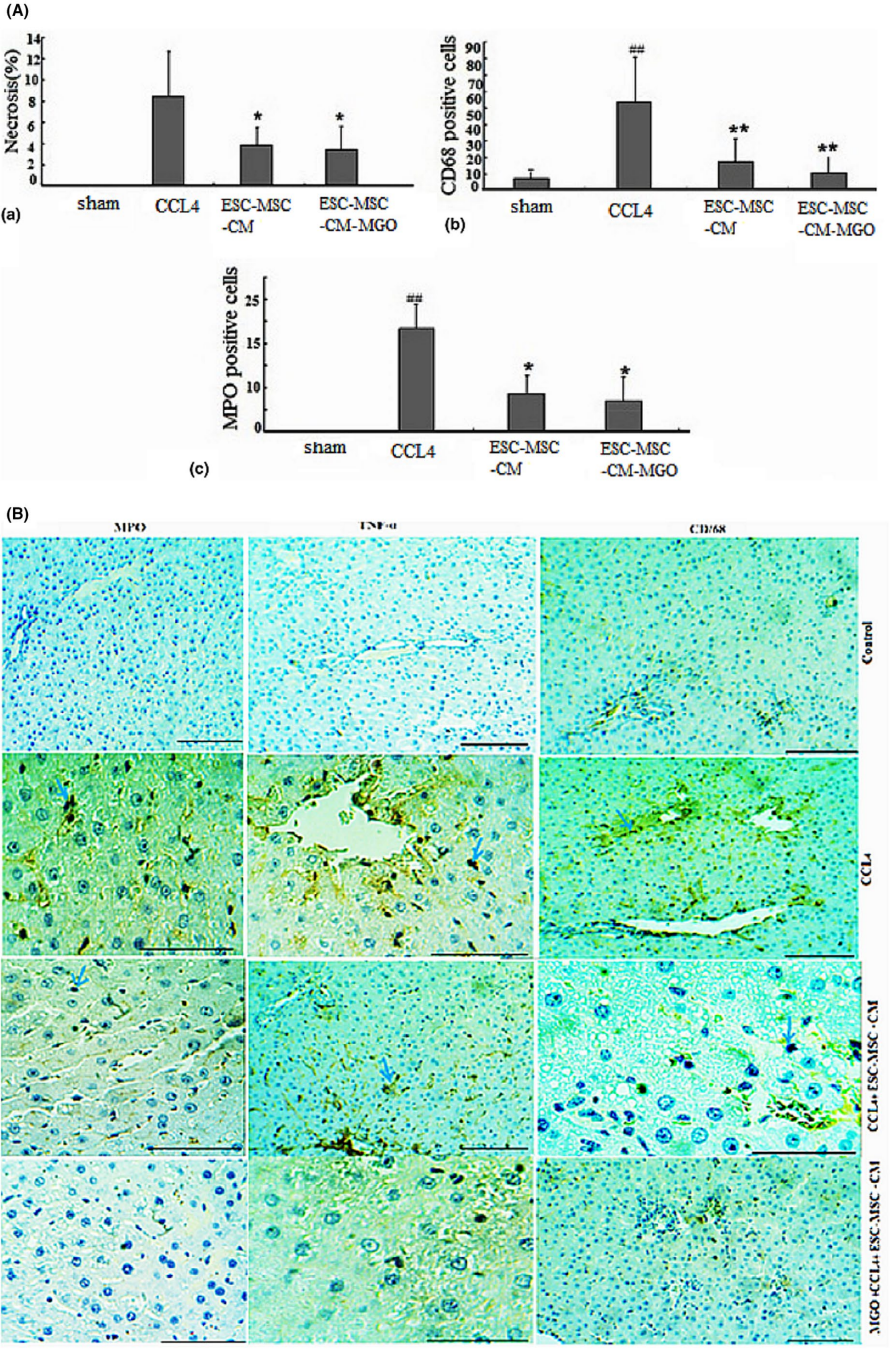
(A) Liver cells necrosis (a) was quantified in the control, CCl_4_, ESC‐MSC‐CM and ESC MSC‐CM + MGO groups. Treatment with MGO reduced infiltration of neutrophils and activation of Kupffer cells, Kupffer cell activation was assessed by labelling CD68 (b) in sections 96 h after operation, and neutrophil infiltration was visualized by MPO immunostaining (c) in sections 96 h after operation. *p < 0.05, **p < 0.01; ^##^ *p* < 0.01. (**B**) Treatment with MSC‐CM mixed with MGO improved microscopic histopathologic parameters of ${\mathrm{CCl}}_{4}^{‐}$induced liver damage and reduced infiltration of neutrophils, proinflammatory cytokine TNF‐α, and activation of Kupffer cells. Liver samples taken 4 days after the injection of CCl_4_, ${\mathrm{CCl}}_{4}^{+}$ MGO, ${\mathrm{CCl}}_{4}^{+}$ MSC‐CM and ${\mathrm{CCl}}_{4}^{+}$ MSC‐CM + MGO subject to immunohistologic analysis. Neutrophil, necrosis and Kupffer cell were stained by labelling MPO, TNF‐α and CD68, respectively, in livers sections. Arrows mark MPO^+^, TNF‐α+ and CD68^+^ cells. Abbreviations: CM, conditioned medium; CCl_4_, carbon tetrachloride; MGO, graphene oxide

**FIGURE 4:**
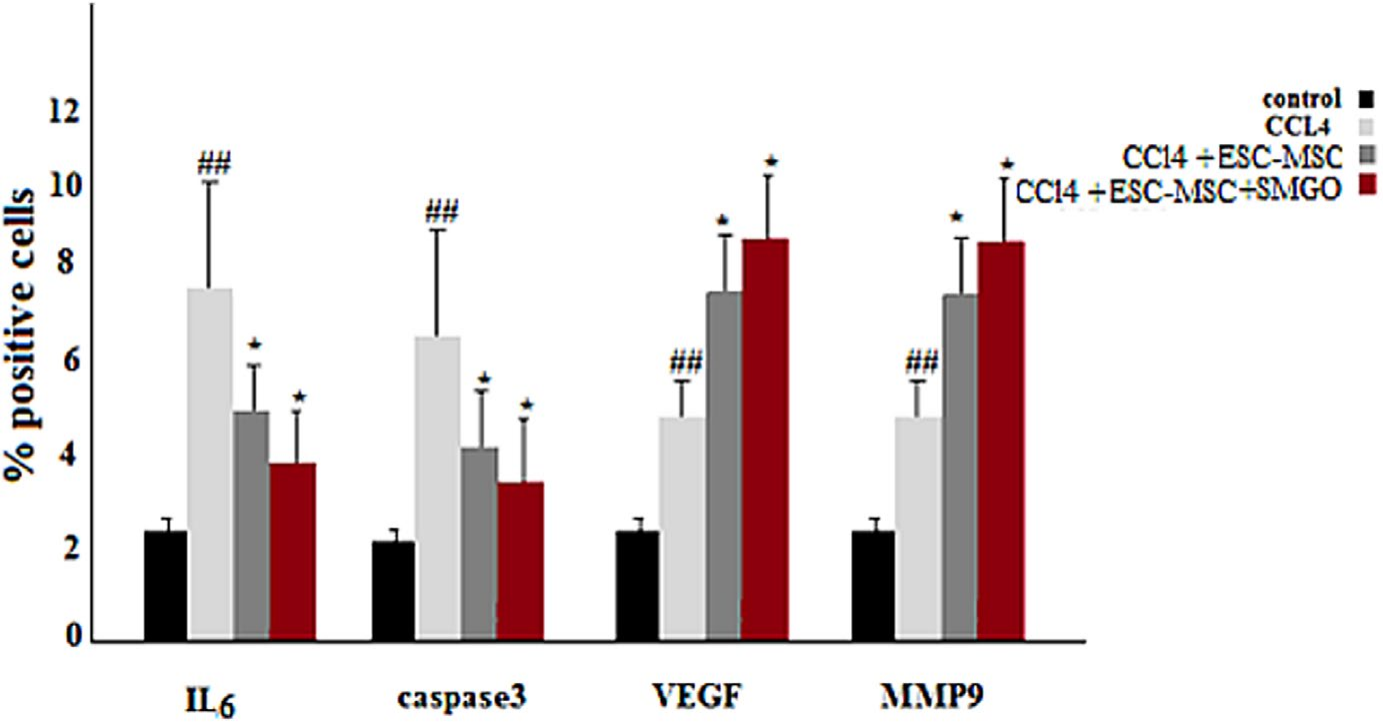
The number of IL‐6 and Caspase‐3 positive cells were significantly lower in the ESC‐ MSC‐CM and MSC‐CM + MGO groups compared to the CCl_4_ group 4 days after the injection. Here, nano refers to MGO. The number of VEGF and MMP‐9 positive cells was significantly higher in the MSC‐CM and MSC‐CM + MGO groups compared to that in the CCl_4_ group (*p < 0.05, ^##^ *p* < 0.01). Abbreviations: CCl_4_, carbon tetrachloride; MGO, magnetic graphene oxide

**FIGURE 5:**
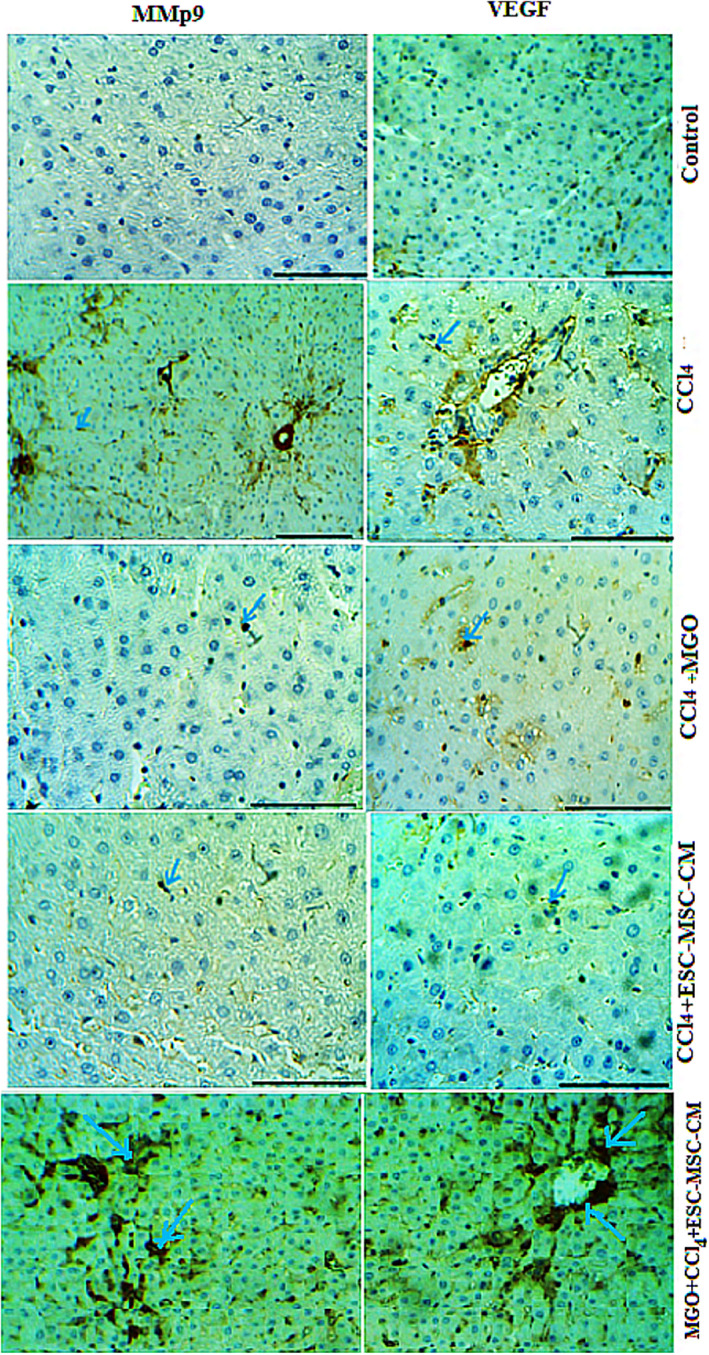
Effects of MGO on the expression of VEGF and MMP‐9 in the damaged livers treated with ESC‐MSC‐CM. MGO enhanced the number of VEGF and MMP‐9‐positive cells in the CCl4‐induced livers treated by MSC‐CM. Positive cells was visualized by VEGF and MMP‐9 immunostaining in the liver sections 4 days after the injection. Scale bar, 200 μm (control, CCl_4_ + MGO and CCl_4_ + CM + MGO); scale bar, 400 μm (CCl_4_, ${\mathrm{CCl}}_{4}^{+}$CM). Abbreviations: CM, conditioned medium; CCL4, carbon tetrachloride; MGO, magnetic graphene oxide

**FIGURE 6:**
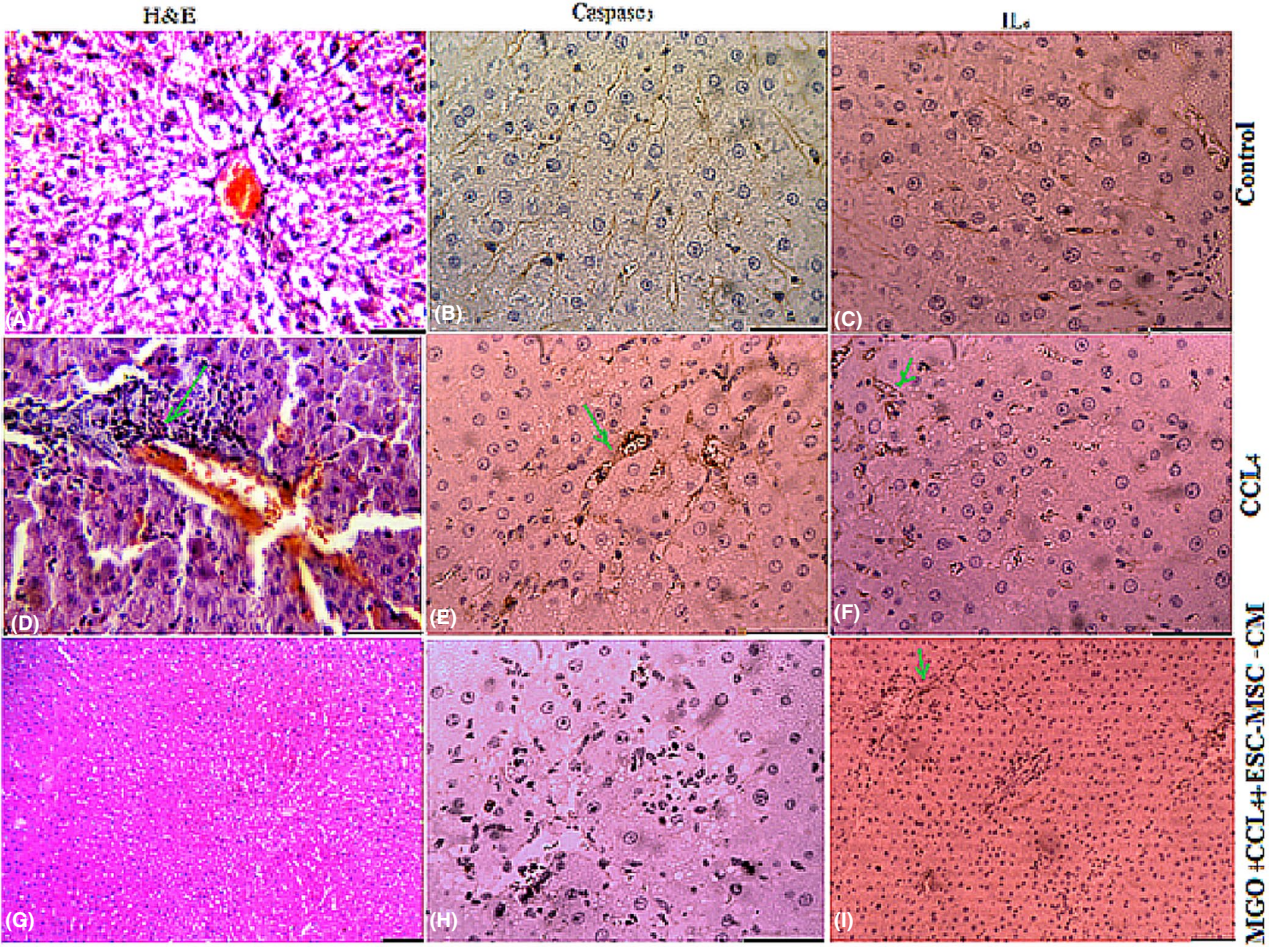
Treatment with ESC‐MSC‐CM + MGO after 3 days decreased the levels of proinflammatory cytokines IL‐6 and Caspase‐3 in liver failure induced by CCl_4_ compared to treatment with ESC‐MSC‐CM‐MGO. Sections stained with H&E, IL‐6 and Caspase‐3. Abbreviations: CM, conditioned medium; CCl_4_, carbon tetrachloride; MGO, magnetic graphene oxide

We also examined the histopathologic changes in H&E and immunostained liver sections (Figures 3, 4, 5, 6, Table 3). In the CCl_4_ group, a significant increase in the number of neutrophil and Kupffer cells was found and compared to the control group. However, only a slight infiltration of inflammatory cell was observed in the ESC‐MSC‐CM + MGO group compared to the ESC‐MSC‐CM and MGO groups (Figures 3, 4, 5, 6). The results indicated a significant reduction in the number of neutrophil and Kupffer cell infiltrations in the MGO and ESC‐MSC‐CM groups compared to the CCl_4_ group (*p* < 0.01) (Figures 3,4). In other words, the number of these cells in the ESC‐MSC‐CM + MGO group was lower than that in the MGO and ESC‐MSC‐CM groups (*p* < 0.01) (Figure 3a). The number of CD68 cells was also significantly lower in the ESC‐MSC‐CM + MGO group compared to other groups (Figure 3b). MPO‐positive cells were also evident in all groups; however, the number of MPO‐positive cells was significantly lower in the CCl_4_ group after treatment with ESC‐MSC‐CM and MGO (*p* < 0.05) (Figure 3c). Treatment with MGO reduced the number of CD68 and MPO‐positive cells in the CCl_4_ESC‐MSC‐CM group significantly (*p* < 0.01). The results indicated that MGO improved the effects of ESC‐MSC‐CM on the treatment of damaged liver.

**TABLE 3:** Microscopic evaluation of hepatocytes in different treatment groups. Treatment with MGO improved the effects of ESC‐MSC‐CM on histopathologic appearance

Hyperemia	Accumulation of inflammatory cells	% Apoptosis	Average apoptotic cells per field	Sample
−	−	0	−	Control
−	−	0	−	Sham
+++	+++	17.71	62	CCl_4_
++	++	5.90	19	MGO
+	+	8.32	29.8	ESC‐MSC‐CM
++	++	0.94	6	ESC‐MSC‐ CM+MGO

Note

−, +, ++, and +++ were used to assess hyperemia and accumulation of inflammatory cells and indicate no effect, slight, mild, and intensive, respectively.

### 3.4 MGO lowers the level of proinflammatory cytokines and increases the expression of vascular endothelial growth factor and MMP‐9 in damaged livers

**FIGURE 7:**
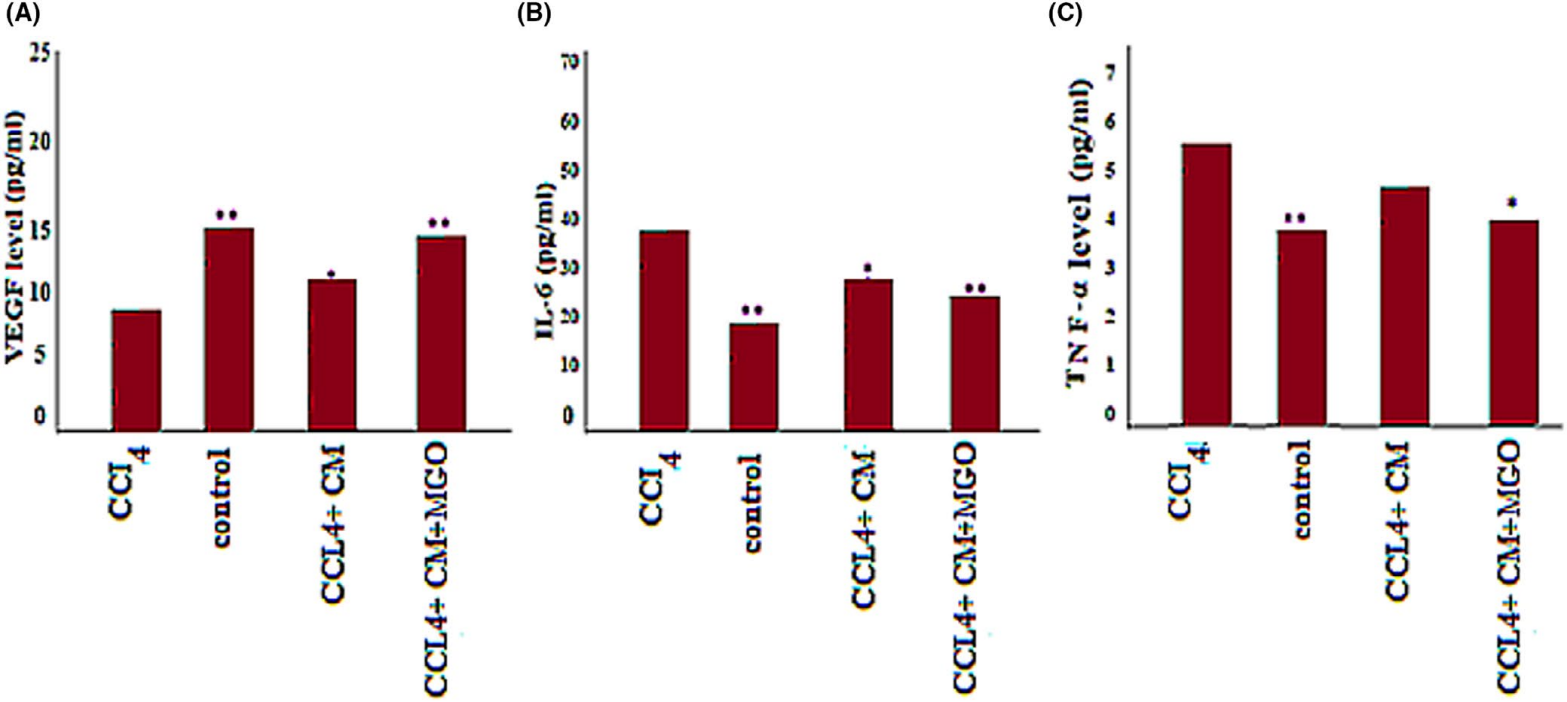
ELISA results of VEG (a)F, IL‐6 (b) and TNF‐ α (c) serum levels in ${\mathrm{CCl}}_{4}^{‐}$ induced rats receiving MGO, MSC‐CM and MGO + ESC‐MSC‐CM. Significant difference between the CCl_4_ and the other groups were *p< 0/05 and **p < 0.001 4 days after the injection. Abbreviations: CM, conditioned medium; CCl_4_, carbon tetrachloride; MGO, magnetic graphene oxide

In the CCl_4_‐induced livers, the expression levels of proinflammatory cytokines such as TNF‐α, IL‐6 and Caspase‐3 decreased after MGO injection. Damaged livers stained with TNF‐α, IL‐6 and Caspase‐3 antibodies showed that MGO injection inhibited apoptosis and necrosis of hepatocytes. Many apoptotic hepatocyte nuclei were observed in the CCl_4_ group. Therefore, MGO promotes the survival of hepatocytes in the CCl_4_ group. The levels of these cytokines were lower in the ESC‐MSC‐CM + MGO group than in the MGO and ESC‐MSC‐CM groups. Moreover, the expression of VEGF and MMP‐9 was significantly upregulated after treatment with MGO (Figure 5). Also ELISA results showed a higher level of VEGF in MGO + ESC‐MSC‐CM compared with CCl_4_ group. On the other hand, higher levels of VEGF were found in MGO + ESC‐MSC‐CM compared with MGO and ESC‐MSC‐CM groups (Figure 7). ELISA results showed a higher level of VEGF in MGO + ESC‐MSC‐CM compared with CCl_4_ group. TNF‐α and IL‐6 expression were lower in the serum of rats receiving MGO compared with ESC‐MSC‐CM group (Figure 7).

## 4 DISCUSSION

The ideal treatment for the CCl_4_‐induced liver damage is to reduce symptoms such as apoptosis and inflammation and to promote immediate liver regeneration. In this study, we have shown that intraperitoneal injection of mixed with ESC‐MSCs condition medium provides a significant survival benefit in rats, followed by a reduction in damage and serum enzymes. Previously, it has been reported that that low doses of GO derivatives (3–8 mg/kg) nm) showed no obvious clinical toxic signs.^65^ Since stem cell restoration of damaged organs is viewed as a paracrine effect rather than the direct effect of MSC entering into the organ, we studied the effects of hESC‐MSC condition medium transplantation on the improvement of the damaged liver.^66^ Van Poll et al. and Parekkadan et al.^67^ showed that MSC‐CM transplantation in rats with liver failure provides a significant survival benefit, as MSC‐CM provides trophic support to the damaged liver by inhibiting hepatocellular death and stimulating regeneration. On the other hand, some studies have reported no survival benefit in animals treated with MSC‐CM and ESC‐MSC‐CM.^15^ Lotfinia et al.^15^ reported, however, that while ESC‐MSC and MSC‐CM improved the function of the damaged liver, they did not increase survival. Several studies have shown antiapoptotic effects of MSC‐CM on hepatocytes using *in vitro* and *in vivo* assays.^66^

Some of the factors secreted from ESC‐MSCs, such as vascular endothelial growth factor, have higher expressions compared to adult MSCs. VEGF is associated with biological processes such as regulation of epithelial cell proliferation and the immune system, as well as negative regulation of the apoptotic process.^15^ Our results showed that the expression of VEGF and MMP‐9 was upregulated 48 h after the injection of the ESC‐MSCs mixed with MGO. Since the addition of MGO to ESC‐MSC‐CM decreased the number of apoptotic cells, we propose that both chemokines, i.e., VEGF within ESC‐MSC‐CM and MGO, play a vital role in treating the damaged liver. Ma et al. showed that stem cells prevent liver apoptosis by secreting VEGF *in vitro*.^68^ They also showed that the ESC‐MSC‐CM is more effective compared to MSC‐CM. Recently, various studies have shown that ESCs‐MSCs possess more constant regenerative medicine effects, compared to adult MSCs.^69^ In other words, embryonic‐MSC lines had consistently superior efficacy over MSCs derived from bone marrow samples. It could be correlated with the lower secretion of the IL6 from the ESCs‐MSCs than from the adult‐MSCs.^63^ They secrete more anti‐inflammation and antiapoptosis factors, attenuating the inflammation and also cardiomyopathy symptoms.^70^ These results confirm the findings of Jiang et al.^63^ They showed that transplantation of ESC‐MSCs has a stronger effect in organ regeneration than that of adult‐MSCs.^71^

However, we observed in our study that ESC‐MSCs mixed with MGO exhibited better anti‐inflammatory and anti‐apoptosis properties *in vivo* than BM‐MSCs, which may be due to the presence of highly expressed mediators in ESC‐MSCs that included VEGF, MPO and MMP9. They have been shown to reduce inflammation^72^ or play a central role in positive regulation of the immune system.^15^ Embryonic stem cells‐MSCs are less mature than bone marrow MSCs, and its conditioned medium has higher paracrine factors with early embryogenesis and angiogenesis property than that by bone marrow‐MSCs.^69^The use of MGO was an effective way to enhance the therapeutic effects of stem cells condition medium. In this study, the positive effects of ESC‐MSC‐CM on the damaged tissue may be due to the physiochemical properties of S MGO or the improved delivery of growth factors within the stem cell medium to the damaged tissue. In other words, although the condition medium improved the survival and increased secretory proteins with trophic and immunomodulatory effects, its effects were less than those of MGO mixed with the medium. This could be due to the effects of the nanodeposition on the surface of trophic factors in serum followed by ECM protein absorption, which is essential for the adhesion, differentiation and growth of hepatocytes. Nevertheless, it could also be due to the capacity of MGO with condition medium to alter the migration of immune cell into the damaged liver.^21^ In addition to its superior mechanical properties, internalization and intracellular fate, GO has a large surface area that makes it suitable for drug delivery and regenerative medicine.^47^, ^73^, ^74^, ^75^, ^76^, ^77^, ^78^, ^79^, ^80^

The spontaneous differentiation induction of MSCs without extrinsic biochemical manipulation is another property of GO.^81^, ^82^ Therefore, in addition to the anti‐apoptotic and proliferative effects of ESC‐MSCs condition medium, the MGO used in the present study can increase the differentiation of liver stem cells into hepatocytes. Indeed, MGO acts as a potential candidate for the delivery of cytokines and growth factors to damaged tissue *in vivo*. Superparamagnetic iron oxide nanoparticles used in the synthesis of MGO facilitate the material transport property through the noncovalent binding of the cytokines and growth factors to GO.^74^, ^76^, ^83^, ^84^

In this work, we synthesized MGO and examined its role in increasing proliferation, angiogenesis and decreasing apoptosis in the damaged liver cells through the ability to bind and release growth factors. We proposed MGO nanoparticles as a new multifunctional target platform for effective delivery of biomolecules capable of treating damaged liver *in vivo*. We showed that GO in combination with Fe_3_O_4_ nanoparticles as biocompatible magnetic material accelerates the delivery of growth factors to the damaged tissue. One of the involved mechanisms is that graphene derivatives strongly adhered to macromolecules such as growth factors, which act as cell‐adhesion substrate and growth factor‐delivery carrier, thereby helps in the regeneration and improving damaged organs.^47^On the other hand, ESC‐MSCs induce a higher rate of hepatocyte proliferation and have stronger anti‐inflammatory properties than bone marrow MSCs. Although MSC‐CM and ESC‐MSC improve liver function, they do not increase survival.^16^ our results showed that MGO can improve the survival from CCl_4_‐induced liver failure in rats received ESC‐MSC‐CM.

## 5 CONCLUSION

Our results indicated that MGO improved the effects of ESC‐MSC‐CM on the treatment of CCl_4_‐induced damaged livers. It inhibited liver parenchymal cell death and improved its regeneration through trophic support, thereby improving survival rate. In addition to stem cell therapy, stem cell modifications or MSCs in combination with other treatment methods are increasingly being considered. We concluded that in addition to transplanting ESC‐MSC‐CM and MSC‐CM, liver damage can be synergistically treated by MGO through preventing apoptosis, enhancing angiogenesis and/or blocking the action of inflammatory factors. We concluded that MGO enhances the therapeutic effects of secreted molecules of embryonic stem cell‐derived mesenchymal stem cells on acute hepatic failure model.

## CONFLICT OF INTEREST

The authors declare that they have no competing interests.

## AUTHOR CONTRIBUTIONS

Tahereh Foroutan designed the study; Mohammad Zaman Kassaee and Mehdi Salari performed most of nanomaterials experiments; Fatemeh Ahmadi and Fatemeh Molavi fed the animals and was involved in preparing of revised manuscript, and Fariborz Moayer assisted in histological study.

## Data Availability

The data that support the findings of this study are available within the manuscript or available from the authors upon request.
